# Immune Checkpoints and Graves' Disease, Thyroid Eye Disease, and Orbital Myopathy: A Comprehensive Review

**DOI:** 10.18502/jovr.v19i3.15047

**Published:** 2024-09-16

**Authors:** Zahra Souri, Farzad Pakdel

**Affiliations:** ^1^Department of Cell and Molecular Biology and Microbiology, Faculty of Biological Science and Technology, University of Isfahan, Isfahan, Iran; ^2^Department of Oculo-Facial Plastic Surgery, Farabi Eye Hospital, Faculty of Medicine, Tehran University of Medical Sciences, Tehran, Iran

**Keywords:** Autoimmunity, Graves' Disease, Immune Checkpoint Inhibitors, Orbital Inflammation, Orbital Myopathy, Thyroid Eye Disease

## Abstract

Immune checkpoints (ICPs) are essential regulators of the immune system, ensuring a delicate balance between self-tolerance and autoimmune responses. ICP therapy is a rapidly growing cancer treatment strategy that inhibits the interaction between ICPs and their ligands. This biological interaction increases the ability of the immune system in combating cancer. However, in some cases, the use of these agents may lead to immune hyperactivity and, subsequently, autoimmune diseases. Graves' disease (GD), thyroid eye disease (TED), and orbital myopathy are complex autoimmune disorders characterized by the production of autoantibodies. The emergence of these treatment-related adverse events underscore the critical need for a deeper understanding of the immune-checkpoint axis in autoimmune diseases. In this review article, we provide a comprehensive survey of the biological mechanisms of ICPs that are most frequently targeted in cancer therapy, including CTLA-4, PD-1, PDL-1, and LAG3. Furthermore, we investigate the latest scientific findings on the adverse events associated with the inhibition of these ICPs. This paper will particularly focus on the potential risks these complications pose to ocular and orbital tissues, which are a concern in the context of cancer treatment.

##  INTRODUCTION

Immune checkpoints (ICPs) are natural regulatory mechanisms that dampen the immune system's response. Physiologically, they maintain a delicate balance between proinflammatory and anti-inflammatory pathways, thereby preventing chronic inflammation.^[1,2]^ Cancer cells exploit ICPs to evade the immune system's surveillance mechanisms, hence enabling uncontrolled proliferation and metastasis.^[3,4]^ Immune checkpoint inhibitors (ICIs) represent a class of cancer immunotherapy that targets and disables specific ICPs, allowing the immune system to unleash its full potential against cancer cells.^[5]^ Multiple ICPs and their corresponding ligands have been identified as potential targets in the fight against cancer, yielding substantial advancements in treatment outcomes and sustained remissions.^[3,6,7,8]^ While ICIs have revolutionized cancer care by enhancing patient survival, they also carry a risk of immune-related adverse events (IRAEs). These autoimmune reactions can occur in any tissue or organ, highlighting the need for careful monitoring and management to mitigate their impact.^[9,10]^ The timing and duration of IRAEs following the use of ICIs are not yet fully understood. The onset of IRAEs can be at any time, making it challenging to anticipate their development. Moreover, the persistence of IRAEs can vary significantly, ranging from rapid resolution after discontinuation of ICIs to prolonged or even chronic conditions that can last for months or longer.^[11,12,13,14]^

**Figure 1:**
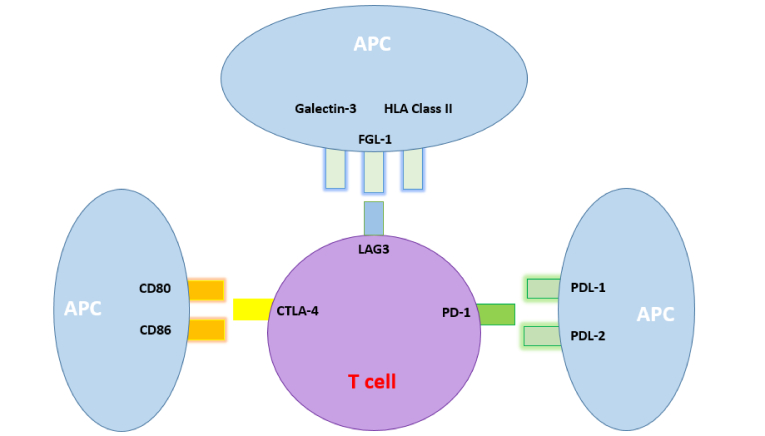
A diagram illustrating the interaction between three immune checkpoint molecules (CTLA-4, LAG3, and PD-1) and their corresponding ligands. The diagram features a T-cell to exemplify an immune cell that carries checkpoint proteins. It also highlights the binding of CTLA-4 to CD80 and CD86; LAG3 to HLA Class II, Galectin-3, and FGL-1; and PD-1 to PDL-1 and PDL-2.

IRAEs can be classified into different categories: endocrine, rheumatological, gastrointestinal, pulmonary, cardiovascular, and neurological.^[5]^ Thyroid dysfunction is considered as one of the most common endocrine IRAEs which may result in clinical symptoms within weeks to months after ICI therapy.^[15,16,17]^ Several studies have revealed a significant association between ICIs and an elevated risk of developing thyroid dysfunction and orbital inflammatory conditions.^[1,2]^ Phase III clinical trials report up to 20% thyroid dysfunction after targeting these cell surface proteins.^[18,19]^ These receptors bind to their respective ligands, transmitting inhibitory signals that ultimately influence the proliferation and differentiation of immune cells.^[20]^ Figure 1 illustrates the interaction between these molecules and their corresponding ligands.

In this comprehensive review, we delve into the biological mechanisms underlying the most frequently targeted ICPs in cancer therapy, including CTLA-4, PD-1, PDL-1, and LAG3. We also examine the latest scientific discoveries on the adverse events associated with the inhibition of these immune modulators with a special emphasis on the potential risks they pose to ocular and orbital tissues. This is particularly important in the context of cancer treatment, where the potential impact on these sensitive areas is a growing concern.

### Overview of the Role of Immune Checkpoints (ICPs) 

#### Cytotoxic T lymphocyte antigen 4 (CTLA-4)

**Cytotoxic T-lymphocyte antigen 4 (CTLA-4) is a member of the immunoglobulin family and consists of 223-amino-acids. This immune modulator is mainly expressed on activated lymphocytes.^[21,22]^ The negative role of CTLA-4 on T-cell proliferation was confirmed when some researchers observed the rapid development of lymphoproliferative disease with multi-organ lymphocytic infiltration and tissue destruction in CTLA-4-deficient mice. These mice developed severe myocarditis and pancreatitis, and they died by three to four weeks of age.^[23]^ Mutation studies on mice have shown that CTLA-4 strongly competes with the co-stimulatory factor CD28 for binding to CD80 and CD86 at immunological synapses. CTLA-4 inhibits the proliferation of T lymphocytes and dampens their cytokine production through binding to CD80 and CD86.^[24,25,26]^ It should be noted that although CTLA-4 plays a significant role in immune regulation, alternative processes ensure immune tolerance when CTLA-4 is absent.^[27,28,29]^**

#### Programmed cell death protein 1 (PD-1)

**Programmed cell death protein 1 (PD-1) is a transmembrane protein composed of 288 amino acids with a critical role in regulating the immune system.^[30]^ This immune modulator is expressed on B-cells, T-cells, as well as NK cells, and it is structurally similar to CTLA-4. PD-1 maintains immune hemostasis by binding to two ligands: programmed cell death ligand 1 (PDL-1) and programmed cell death ligand 2 (PDL-2). PDL-1 is a member of the B7 family and, by binding to PD-1, inhibits lymphocyte proliferation, cytokine production, and CD28 co-stimulation. This factor has shown to be upregulated on peripheral blood mononuclear cells by interferon-gamma stimulation. Additionally, it could be expressed by non-lymphoid tissues such as those making up the heart and lung. PD-L1 expression on non-lymphoid tissues and its potential interaction with PD-1 may subsequently determine the extent of immune responses at sites of inflammation.^[30]^ PDL-2 is a protein that can bind to the PD-1 receptor on T-cells. The role of PDL-2 in regulating the immune system is not well-understood. Its expression depends on Toll-like receptor 4 and STAT1 (signal transducer and activator of transcription 1) factors. PDL-2 is not expressed on inflammatory macrophages but can be induced on them by interleukin 4 stimulation. The level of PDL-2 expression is mediated by both interleukin 4 receptor alpha and STAT6 factors.^[31,32]^**

#### Lymphocyte-activation gene 3 (LAG3)

Lymphocyte-activation gene 3 (LAG3), a member of the immunoglobulin superfamily, is initially expressed at undetectable levels in quiescent peripheral blood lymphocytes. However, upon activation, its expression is upregulated in both T-cells and natural killer cells. The ICP molecule LAG3 is also found on activated B cells, albeit at a lower level.^[33]^ This discovery suggests that LAG3 may play a distinct role in regulating B cell function in addition to its established role in T-cell regulation.^[33,34,35]^ Research findings demonstrate that interactions between LAG3 and HLA Class II downregulate CD4 T-cell activity.^[36]^

### Immune Checkpoint Inhibition (ICI) and Immune-related Adverse Events (IRAEs)

Over the past decade, ICIs have emerged as a groundbreaking treatment approach for various types of cancer, bringing renewed optimism and improved outcomes for patients. The breakthrough began with the FDA approval of ipilimumab in 2011 as the first instance of ICI in cancer therapy. Cancer cells exploit ICPs to avoid detection and attack by the immune system. Neoplastic cells evade immune surveillance by exhausting cytotoxic T-cells. This exhaustion results from prolonged exposure to tumor antigens, which ultimately impairs T-cell functionality and triggers the upregulation of ICP molecules.^[45,46]^ The CTLA-4 inhibitor; ipilimumab; PD-1 inhibitors nivolumab, pembrolizumab, and cemiplimab; and PD-L1 inhibitors atezolizumab, avelumab, and durvalumab are recent FDA-approved antibodies that have been tested either alone or in combination in different trials. Recently, several new ICPs such as BTLA, VISTA, TIM-3, LAG3, and CD47 and co-stimulatory molecules such as CD137, OX40, and GITR have been identified as new immune treatment targets for different cancers.^[47]^ By blocking these checkpoints, ICIs enable the immune system to recognize and destroy cancer cells more effectively, leading to improved survival rates for patients with different types of cancer.^[48]^ The effectiveness of ICIs in managing multiple types of cancer and improving survival rates has led to their widespread use in treatment.^[49,50]^ However, while these treatments have shown positive results, they have also led to uncontrolled immune-related responses that affect various organs. This has become a growing concern in the administration of ICIs because they can cause inflammation and damage healthy tissues.^[51,52,53]^ According to research, a substantial number of patients treated with immunotherapy experience severe adverse events, with frequencies of 14% for anti-PD-L1 inhibitors, 34% for anti-CTLA-4 treatment, and an alarming 55% for combination therapy involving ICIs.^[54]^ IRAEs arise when the immune system mistakenly attacks healthy cells and tissues, resulting in significant inflammation and damage. These events range from mild symptoms such as fatigue and fever to more severe manifestations including colitis, hepatitis, and pneumonitis. In some cases, they can be life-threatening and necessitate hospitalization or discontinuation of treatment.^[9,12,55]^

Although nivolumab and pembrolizumab have shown to be effective in treating certain types of cancer, both drugs also carry the risk of potential adverse reactions, particularly thyroiditis, thyroid eye disease (TED), and orbital inflammation.^[56,57,58,59,60,61,62,63,64,65,66,67]^

Given the high rate of severe adverse events associated with immunotherapy, it is crucial to better understand the underlying mechanisms driving these reactions and implement rigorous monitoring strategies to ensure patient safety throughout the treatment process. In the following section, we address the orbital and thyroid-specific adverse events associated with ICIs, with a particular emphasis on the development of thyroid autoimmune dysfunction and orbital-related inflammatory responses [Table 1].

### Immune Checkpoint Inhibition (ICI) and Thyroid Immune-related Adverse Events (IRAEs)

Side effects that occur in the thyroid gland as a result of treatment with ICIs are reffered to as thyroid IRAEs. Recent research has demonstrated the effectiveness of combining anti-CTLA-4 and anti-PDL-1 treatments for advanced cancers. It is crucial to recognize that, with an increasing number of patients receiving these treatments, there has been a growing incidence of autoimmune-related endocrine disorders. Reports on ICIs-associated thyroid and orbital inflammations have been summarized in Table 1.

Given that reports show thyroid IRAEs frequently occur after therapy with ICIs, it is important to investigate the molecular mechanisms that are responsible for such complications to identify the patients who are at risk for thyroid IRAEs.

### Immune Checkpoint Inhibition (ICI) and Graves' Disease (GD)

Graves' disease (GD) is a chronic autoimmune disorder characterized by the production of antibodies that target the thyroid gland, orbital soft tissues, and skin, leading to thyroid dysfunction, distinctive orbital and skin manifestations, and a range of associated symptoms.^[68,69]^ Thyroid-stimulating hormone-binding inhibitory immunoglobulins (TBII) are autoantibodies which act against the thyroid-stimulating hormone receptor in response to hyperthyroidism and lead to the pathogenesis observed in GD.^[70]^

Researchers have identified genetic variations in the *CTLA-4* gene that are associated with an increased activity of T-cells, and can lead to the development of autoimmune disorders such as GD and autoimmune hypothyroidism. They found a correlation between susceptibility to these diseases and a 6.1-kb region of the *CTLA-4* gene that results in lower levels of messenger RNA (mRNA) for the alternative soluble splice form of the CTLA-4 protein. The lower levels of this specific form of CTLA-4 mRNA may contribute to increased susceptibility to GD and autoimmune hypothyroidism.^[71]^ A study identified CTLA-4 as a potential factor contributing to the development of GD in the Chinese population. The authors investigated the association between two CTLA-4 polymorphisms (+49A/G and CT60) and GD as well as TED, also referred as Graves' orbitopathy. The meta-analysis revealed that both polymorphisms were linked to GD, but no significant association was found between these polymorphisms and TED in patients with GD. It was suggested that the +49A-CT60G haplotype may increase the risk of TED in patients with GD, with an odds ratio (OR) of 1.63 and a 95% confidence interval (CI) of 1.00–2.64; nevertheless, this association was marginally significant (*P* = 0.05).^[72]^ A team of researchers attempted to establish a correlation between variations in the *CTLA-4* gene and the likelihood of developing GD. A total of 329 patients with GD (240 of whom were positive for TBII and 89 were negative for TBII) and 378 healthy individuals (as controls) were studied for genetic variations in the *HLA-A*, –*DPB1*, and *CTLA4* genes. In this study, individuals with GD who tested positive for TBII had a higher incidence of TED compared with the controls (97.1% vs 91.8%; OR = 2.97, 95% CI = 1.29–6.87, *P *= 0.008). However, there were no significant differences in the TED incidence between TBII-negative patients with GD and the controls (94.4% vs 91.8%; OR = 1.50, 95% CI = 0.57–3.98, *P* = 0.41). This study reported on an association between the *CTLA-4* gene and susceptibility to TBII-positive GD.^[73]^ A 51-year-old woman with lung melanoma and skin tumors was treated with ipilimumab. After just two treatments, she began to exhibit GD symptoms, including severe eye bulging, double vision, and dry eyes, which were caused by inflammation of the eye muscles. Further investigation revealed that the activation and proliferation of T-cells following therapy with this ICI were linked to the production of cytokines, highlighting the importance of the CTLA-4 receptor in the development of this autoimmune disorder.^[74]^

In a case report, it was observed that a 67-year-old euthyroid male patient with metastatic melanoma developed signs of hyperthyroidism after two of four scheduled cycles of therapy with ipilimumab. Upon diagnosis, the patient was found to have developed GD. As a result, the anti-CTLA-4 therapy was discontinued and his thyroid function was restored through treatment with methimazole.^[75]^ In a separate study, a 55-year-old man with metastatic skin melanoma was treated with a combination of temozolomide, rucaparib, and tremelimumab, an anti-CTLA-4 agent. Eight years into treatment, he developed GD, highlighting the potential risk of autoimmune thyroiditis when CTLA-4 is targeted through therapy. This case emphasizes the significance of concurrent administration of antithyroid medication alongside CTLA-4 suppression therapy to mitigate the risk of developing autoimmune disorders.^[76]^ A 51-year-old man who was under treatment with nivolumab for metastatic non-small cell lung cancer presented palpitations, heat intolerance, and insomnia after his fourth infusion. Although being euthyroid before treatment with nivolumab, the patient presented symptomatic thyrotoxicosis two months after therapy. Persistence of hyperthyroidism, hypervascular pattern at thyroid ultrasound, and high uptake at thyroid scintigraphy confirmed the development of GD. In order to restore the thyroid function, he underwent therapy with methimazole (20 mg/day) and euthyroidism was restored after 60 days of treatment.^[77]^

Another study reported a 66-year-old individual with HER2-positive stomach cancer. Due to liver metastasis with a portal tumor thrombus, he received the first line of therapy with eight cycles of tegafur/gimeracil/oteracil (S-1), cisplatin, and trastuzumab. Because of disease progression, he underwent the second line of therapy and received paclitaxel and ramucirumab. After 10 cycles, the disease progressed again, and nivolumab was administerd this time. Thyroid-stimulating hormone receptor antibody (TRAb) and thyroid-stimulating antibody (TSAb) tests were negative before the first dose of nivolumab, but became positive after starting the therapy. TSH suppression and thyrotoxicosis occurred before the second and third administrations, respectively. As TRAb and TSAb were positive before the second administration, the onset of GD was confirmed after receiving nivolumab.^[78]^

### Immune Checkpoint Inhibition (ICI) and Thyroid Eye Disease (TED)

Inflammation can arise in the orbital and surrounding orbital tissues and pose a significant risk of serious complications.^[38,79]^ Autoimmune orbital inflammatory diseases are a group of disorders that target the eye and its surrounding tissues. They can lead to loss of vision and other ocular symptoms such as pain, redness, and swelling. The exact cause of orbital inflammation is not fully understood, but it is believed to be related to an autoimmune response.

**Table 1:** Auto- immune Adverse Events in Trials Using Immune Checkpoint Inhibitors.


**Agent**	**Molecular target**	**Tumor**	**Dosage**	**Auto-immune AE**	**Study**
Ipilimumab	CTLA-4	Stage III or IV melanoma	3 mg/kg	Grade III or IV IrAEs	*Hodi 2010 ^[55]^*
	Stage IV melanoma	10 mg/kg	Graves' ophthalmopathy	*Min 2011 ^[58]^*
	Lung malignant melanoma	N/A	T3 depression Graves' disease Graves' ophthalmopathy	*Borodic 2011 ^[74]^*
	Metastatic melanoma	3 or 10 mg/kg	Hypothyroidism-Thyroiditis	*Ryder 2014 ^[61]^*
	Malignant melanoma with liver and lung metastases	3 mg/kg	Thyroid-like ophthalmopathy	*McElnea 2014 ^[99]^*
	Advanced melanoma	3 mg/kg	Grade III-V TRAEs	*Robert 2015 ^[57]^*
	Metastatic Melanoma	3 mg/kg	Hyperthyroidism- Graves' disease	*Azmat 2016 ^[75]^*
Ipilimumab+ Bevacizumab	CTLA-4+ VEGF	Advanced melanoma	10mg/kg+7.5mg/kg	Autoimmune thyroiditis	*Min 2011 ^[58]^*
Tremelimumab	CTLA-4	Skin metastatic melanoma- lung metastases	N/A	Hyperthyroidism- Graves' disease	*Gan 2017 ^[76]^*
	Metastatic cutaneous melanoma	10mg/kg	Hyperthyroidism Graves' disease Graves orbitopathy	*Sagiv 2019 ^[13]^*
BMS-936558	PD-1	Non-small-cell lung cancer, melanoma, or renal-cell cancer	1, 3, or 10 mg/kg	Grade III or IV TRAEs Hyper and Hypothyroidism	*Topalian 2012 ^[59]^*
MDX-11-5	PD-1	Selected Advanced or Recurrent Solid Tumors	10 mg	Grade III or IV immune-related adverse events Hypothyroidism Autoimmune thyroiditis Dry eye Hypersensitivity	*Brahmer 2012 ^[65]^*
Atezolizumab	PDL-1	Metastatic Renal Cell Carcinoma	1-20 mg/kg	Grade I-III IrAEs Hypothyroidism	*McDermott 2016 ^[66]^*
Nivolumab	PD-1	Advanced melanoma	1, 3, or 10 mg/kg	Grade III-IV TRAEs Hyper and Hypothyroidism	*Topalian 2014 ^[60]^*
	Clear-cell mRCC	2mg/kg	Grade III-IV TRAEs	*Motzer 2015 ^[56]^*
	Metastatic melanoma without a BRAF mutation	3 mg/kg	Grade III-IV TRAEs Hyper and Hypothyroidism	*Robert 2015 ^[62]^*
	Melanoma	2 mg/kg	Myasthenia gravis	*Suzuki 2017 ^[107]^*
	Non-Small Cell Lung Cancer	3 mg/kg	Myasthenia gravis	*Suzuki 2017 ^[107]^*
	Metastatic renal cell carcinoma	3 mg/kg	Thyroiditis- Graves' disease- Graves orbitopathy	*Sagiv 2019 ^[13]^*
	Metastatic non-small cell lung cancer	3 mg/kg	Autoimmune hyperthyroidism Graves' disease	*Brancatella 2019 ^[77]^*
	Stage IVb gastric cancer	240 mg	Thyrotoxicosis Graves' disease	*Yamada 2020 ^[78]^*
Nivolumab+ Ipilimumab	PD-1+ CTLA-4	Stage III or IV metastatic melanoma	1 mg/kg+ 3mg/kg	Grade III or IV TRAEs Hyper and Hypothyroidism	*Larkin 2015 ^[63]^*
	Hepatocellular Bladder urothelial carcinoma Lymph nodes and bone metastasis	3mg/kg+ 1mg/kg	Bilateral Graves orbitopathy Orbital inflammation	*Sagiv 2019 ^[13]^*
Cemiplimab	PD-1	Squamous cell carcinoma	350 mg	Myasthenia gravis Myocarditis-Myositis	*Jeyakumar 2020 ^[114]^*
Pembrolizumab	Ipilimumab-refractory Advanced melanoma	2mg/kg	Grade III or IV IrAEs	*Robert 2014 ^[57]^*
	Advanced melanoma	10 mg/kg	Grade III or IV TRAEs Hyper and Hypothyroidism	*Robert 2015 ^[10]^*
	Advanced non-small-cell- lung cancer	10 mg/kg	Grade III-V TRAEs Hyper and Hypothyroidism Infusion-related reactions Pneumonitis	*Garon 2015 ^[64]^*
	Advanced urinary cancer	N/A	Ocular myasthenia gravis	*Kamo 2019 ^[110]^*
	
	
	Lung cancer lymph nodes and brain metastasis	N/A	Ocular myasthenia gravis	*Kamo 2019 ^[110]^*
	Metastatic non-small cell lung cancer	N/A	Idiopathic orbital inflammatory syndrome	*Michels 2019 ^[112]^*
	Acral lentiginous melanoma	N/A	Ocular myasthenia gravis	*Liu 2019 ^[111]^*
	Malignant mesothelioma	N/A	Ocular myasthenia gravis	*Lorenzo 2020 ^[113]^*
	N/A	N/A	Ocular myositis	*Garibaldi 2020 ^[115]^*
	High-grade urothelial carcinoma	200 mg	Myasthenia gravis-like disorder- ocular myositis	*Tian 2021 ^[116]^*
	Gastric adenocarcinoma	2 mg/kg	Ocular myasthenia gravis	*Garcez 2022 ^[118]^*
	
	
AE, Adverse event; IrAEs, Immune-related adverse events; N/A, Not applicable; TRAEs, Treatment related adverse events.					

This response is mediated by macrophages and infiltrating T-cells, which play major roles in initiating and augmenting inflammatory processes by releasing pro-inflammatory cytokines such as IL-1, IL-5, TNF- α , and IFN- γ .^[79,80,81]^ Recent studies have suggested a possible association between ICIs and orbital inflammatory conditions, also known as ophthalmic IRAEs.^[82]^

A recent retrospective study investigated the incidence of IRAEs after therapy with ICIs in a Chinese cohort of 962 patients and reported a frequency of 23.5% for IRAEs and 1.1% for OIRAEs.^[83]^

Both CTLA-4 and PD-L1 ICPs have been associated with inflammatory or autoimmune reactions in the orbit.^[84]^ TED is the most frequent extra-thyroidal manifestation associated with GD.^[85,86,87,88]^ Up to 50% of patients with GD develop autoimmunity in the orbit.^[89,90]^ It occurs most commonly in adults but may also affect children.^[91]^ Symptoms of TED include ocular pain, excessive tearing, photophobia, visual disturbances, eyelid retraction, exophthalmos, restrictive extraocular myopathy, and optic nerve dysfunction.^[92]^ TED may be associated with decreased vision secondary to dysthyroid optic neuropathy or keratopathy.^[93,94,95,96]^ The significant impact of TED on patients' quality of life has been well documented, making it a critical issue that warrants attention and management.^[97,98,99]^ According to the European Group on Graves' Orbitopathy (EUGOGO), three distinct categories exist for this complication: mild, moderate to severe, and sight-threatening.^[85,92]^ In cases where ophthalmic pathological conditions arise, approximately 80–90% of patients will exhibit hyperthyroidism, yet hypothyroidism may also be present. TED is typically characterized by two distinct phases: an active inflammatory phase and a static phase.^[85,93]^ Notably, TED often presents as a bilateral condition, but it can occasionally appear as unilateral or asymmetric.^[100,101]^

It has been shown that the *CTLA-4* gene is associated with the presence of thyroid antibodies (TAbs) and the development of TED.^[90]^ A study analyzed 529 cases and identified a correlation between a single-nucleotide polymorphism (SNP) at position 49 of the *CTLA-4* gene, where an A or G nucleotide can be present, and the onset of autoimmune TED. Specifically, the study found that the G allele is associated with a decrease in CTLA-4 function. The finding suggested that genetic variations in CTLA-4 may contribute to the development of orbitopathy. The authors concluded that further research is needed to confirm these results and elucidate the mechanisms by which CTLA-4 SNPs affect immune system function.^[94]^

Studies propose that immunotherapy with ICIs triggers the production of autoantibodies against thyroid-stimulating hormone receptors. This phenomenon leads to excessive activity of immune cells within the orbit, causing tissue damage and inflammation and contributing to TED pathogenesis.^[95,96]^ A case report focused on a 51-year-old female patient with stage IV melanoma and no history of thyroid disease. The patient showed eye pain, conjunctivitis, and periorbital edema compatible with TED after receiving four doses of ipilimumab 10 mg/kg.^[58]^ Moreover, another study reported a 51-year-old female with lung malignant melanoma who showed TED-like symptoms after two infusions of ipilimumab. This observation suggests the significant role of CTLA-4 in regulating T-cell activities in the development of TED.^[85]^ In accordance with previous findings, another study found that the use of ipilimumab in a euthyroid 68-year-old woman with metastatic melanoma intensified the development of TED symptoms and signs secondary to the treatment including ophthalmoplegia, bilateral enlargement of all extra-ocular muscles, and bilateral proptosis.^[105]^ In a literature review from 1990 to 2017, it was identified that various ocular and orbital side effects are associated with the inhibition of ICPs. These side effects included uveitis, dry eyes, myasthenia gravis, inflammatory orbitopathy similar to thyroid and thyroid-like orbitopathy, keratitis, cranial nerve palsy, optic neuropathy, serous retinal detachment, and neuroretinitis. The study highlighted the importance of monitoring for these side effects when using anti-ICP therapies due to their potential impact on the eye and the surrounding structures.^[106]^ A retrospective study reported that three patients who were treated with ICIs developed TED-like orbital inflammation. One patient was a 73-year-old man with a history of hepatocellular carcinoma who was diagnosed with bladder urothelial carcinoma with metastasis to bone and lymph nodes and was treated with ipilimumab and nivolumab. Despite normal thyroid function (normal TSH, T4, TSI, and anti-TPO), the patient experienced symptoms such as periocular pain, pain with eye movement, ocular irritation, eyelid swelling, erythema, and double vision six weeks after treatment. Ocular inflammatory side effects were resolved after high-dose intravenous steroids. The second patient was a 42-year-old man with metastatic renal cell carcinoma who developed hyperthyroidism and GD after treatment with the multi-tyrosine kinase inhibitor pazopanib. A year later, soon after receiving anti PD-1 nivolumab, he developed bilateral upper eyelid retraction, double vision, medial rectus and inferior rectus muscle enlargement, and other TED-like symptoms. Thyroid tests confirmed low TSH and increased T3, T4, and TSI. Because hypothyroidism was observed, the patient underwent thyroid hormone replacement and his diplopia improved. The third patient was a 51-year-old man with cutaneous metastatic melanoma who developed acute hyperthyroidism (low serum TSH and TSI, increased T3, T4, and anti-TPO) and TED-like symptoms (acute periocular swelling and erythema with bilateral exophthalmos) after therapy with anti-CTLA-4 using the non-FDA-approved tremelimumab agent. This patient was also diagnosed with GD associated with TED and symptomatic inflammation. Because of severe orbital inflammation, he received intravenous steroids followed by oral methylprednisolone, and his orbital inflammation completely resolved after three months of therapy.^[13]^

Research findings highlight the fact that orbital inflammation may be active in patients with TED because of insufficient ant-inflammatory regulation. A study showed that the fibroblasts of patients with TED do not express PDL-1 (measured by flow cytometry). This study evaluated a total of eight patients and compared them with five healthy individuals (controls) who did not have any ocular complaints or issues. T-cells were co-cultured together with fibroblasts and PDL-1 was applied exogenously in order to inhibit T-cell activity. The exogenous expression of PDL-1 resulted in a decrease in T-cell-induced fibroblastic activity, as well as a reduction in the production of several key inflammatory factors, including soluble ICAM-1, IL-6, IL-8, and hyaluronan. (These changes were measured using ELISA assays.) Furthermore, external PDL-1 was found to suppress CD40 expression, which was confirmed through flow cytometry analysis. This inhibition prevented the activation of both the MAPK and NF- κ B signaling pathways in orbital fibroblasts. It was revealed that the suppression of CD40 expression through the use of CD40 siRNA leads to a decrease in the production of IL-6, IL-8, and hyaluronan. Additionally, the inhibition of the phosphorylation of MAPK and NF- κ B pathways through the use of SB203580, PD98059, SP600125, and PDTC explained the reduction in the expression of these molecules. Overall, this study suggested that exogenous PDL-1 administration may be a potential way to reconstruct immune tolerance in TED.^[107]^

### Immune Checkpoint Inhibition (ICI), Ocular Myasthenia Gravis, and Orbital Myositis

Myasthenia gravis (MG) is an autoimmune disorder that affects the communication between nerve cells and muscles. Although this disease is not associated with orbital inflammation, it shows autoimmunity on the neuromuscular junction including extraocular muscles. During the course of the disease, autoantibodies target postsynaptic neuromuscular junctions and prevent normal neuromuscular signal transmission, leading to muscle weakness and fatigue.^[108,109]^ Individuals with MG have a significantly increased risk of developing TED, with a 2.3-fold greater likelihood of developing this condition compared to those without MG.^[110,111]^ Moreover, it has been reported that this disease can coexist in patients with thyroid-associated GD and may even remain undiagnosed because of similar clinical features.^[112,113,114]^ Ophthalmic MG (OMG) is considered a potential IRAE in patients receiving ICIs such as pembrolizumab,^[115]^ hence the importance of close monitoring for ocular symptoms in these patients. In this regard, a study reported that 0.12% of patients diagnosed with cancer had developed MG as a result of nivolumab therapy. The authors recommended monitoring for the potential emergence of this condition following intravenous immunoglobulin therapy.^[116]^ A number of studies have observed the incidence of MG after treatment with pembrolizumab^[118,119,120,121,122,123]^ and cemiplimab.^[124]^ A study reported a patient who had symptoms consistent with paralytic myopathy and orbital myositis and was treated with pembrolizumab and Lenvatinib.^[125]^ Another study reported a patient who developed MG-like ophthalmoplegia and orbital myositis after treatment with pembrolizumab.^[126]^ A separate report described a patient with melanoma who developed bilateral orbital myositis after treatment with ipilimumab and recovered by corticosteroid therapy. ${\;}^{\left[127\right]}$ Although ICIs such as nivolumab may worsen symptoms of MG in individuals with a pre-existing disease, it is crucial to acknowledge that some patients with cancer have exhibited improved responses to this treatment, suggesting that it may not be entirely contraindicated in this population. These observations highlight the importance of considering the potential benefits and risks of nivolumab and other ICIs in patients with MG and cancer, thus allowing for a more nuanced approach to managing this complex patient population.^[116]^

##  CONCLUSION

The use of checkpoint inhibitors for cancer treatment can lead to IRAEs, including GD, TED, myasthenia gravis, and orbital myositis. Healthcare professionals should closely monitor for these adverse events, particularly ophthalmological disorders such as orbitopathy, in patients receiving these medications. Accordingly, there must be appropriate communication and collaboration between oncologists and ophthalmologists in managing patients undergoing immunotherapy. Further research is necessary to understand the long-term effects and potential genetic predispositions associated with such immune-related complications and their management strategy in these patients.

### Financial Support and Sponsorship

None.

### Conflicts of Interest

None.

## References

[B1] Tison A, Garaud S, Chiche L, Cornec D, Kostine M (2022). Immune-checkpoint inhibitor use in patients with cancer and pre-existing autoimmune diseases. Nat Rev Rheumatol.

[B2] Ibis B, Aliazis K, Cao C, Yenyuwadee S, Boussiotis VA (2023). Immune-related adverse effects of checkpoint immunotherapy and implications for the treatment of patients with cancer and autoimmune diseases. Front Immunol.

[B3] Ribas A, Wolchok JD (2018). Cancer immunotherapy using checkpoint blockade. Science.

[B4] Souri Z, Wierenga AP, Kroes WG, van der Velden PA, Verdijk RM, Eikmans M, et al (2021). LAG3 and its ligands show increased expression in high-risk uveal melanoma. Cancers (Basel).

[B5] Johnson DB, Nebhan CA, Moslehi JJ, Balko JM (2022). Immune-checkpoint inhibitors: Long-term implications of toxicity. Nat Rev Clin Oncol.

[B6] Yokoyama R, Sato Y, Nakamura F, Kagemoto K, Mitsui Y, Okamoto K, et al (2023). Efficacy of immune checkpoint inhibitors in patients with anorectal melanoma in association with immune-related adverse events: A case series. Clin J Gastroenterol.

[B7] Thomas B, Burns M, Pervanas H, Ciurescu D, Dima L (2023). Nivolumab/relatlimab-rmbw: A novel dual combination therapy to treat adult and pediatric patients with unresectable or metastatic melanoma. Am J Ther.

[B8] Huber RM (2024). Neoadjuvant therapy with immune checkpoint inhibitors in operable nonsmall cell lung cancer. Curr Opin Oncol.

[B9] Esfahani K, Meti N, Miller WH Jr, Hudson M (2019). Adverse events associated with immune checkpoint inhibitor treatment for cancer. CMAJ.

[B10] Robert C, Schachter J, Long GV, Arance A, Grob JJ, Mortier L, et al (2015). ; KEYNOTE-006 investigators. Pembrolizumab versus ipilimumab in advanced melanoma N Engl J Med.

[B11] Weber JS, Kähler KC, Hauschild A (2012). Management of immune-related adverse events and kinetics of response with ipilimumab. J Clin Oncol.

[B12] Owen CN, Bai X, Quah T, Lo SN, Allayous C, Callaghan S, et al (2021). Delayed immune-related adverse events with anti-PD-1-based immunotherapy in melanoma. Ann Oncol.

[B13] Sagiv O, Kandl TJ, Thakar SD, Thuro BA, Busaidy NL, Cabanillas M, et al (2019). Extraocular muscle enlargement and thyroid eye disease-like orbital inflammation associated with immune checkpoint inhibitor therapy in cancer patients. Ophthalmic Plast Reconstr Surg.

[B14] Horisberger K, Portenkirchner C, Rickenbacher A, Biedermann L, Gubler C, Turina M (2021). Long-term immune-related adverse events after discontinuation of immunotherapy. Immunotherapy.

[B15] Byun DJ, Wolchok JD, Rosenberg LM, Girotra M (2017). Cancer immunotherapy - Immune checkpoint blockade and associated endocrinopathies. Nat Rev Endocrinol.

[B16] Barroso-Sousa R, Barry WT, Garrido-Castro AC, Hodi FS, Min L, Krop IE, et al (2018). Incidence of endocrine dysfunction following the use of different immune checkpoint inhibitor regimens: A systematic review and meta-analysis. JAMA Oncol.

[B17] Muir CA, Clifton-Bligh RJ, Long GV, Scolyer RA, Lo SN, Carlino MS, et al (2021). Thyroid immune-related adverse events following immune checkpoint inhibitor treatment. J Clin Endocrinol Metab.

[B18] Larkin J, Chiarion-Sileni V, Gonzalez R, Grob JJ, Rutkowski P, Lao CD, et al (2019). Five-year survival with combined nivolumab and ipilimumab in advanced melanoma. N Engl J Med.

[B19] de Filette J, Andreescu CE, Cools F, Bravenboer B, Velkeniers B (2019). A systematic review and meta-analysis of endocrine-related adverse events associated with immune checkpoint inhibitors. Horm Metab Res.

[B20] Dyck L, Mills KH (2017). Immune checkpoints and their inhibition in cancer and infectious diseases. Eur J Immunol.

[B21] Brunet JF, Denizot F, Luciani MF, Roux-Dosseto M, Suzan M, Mattei MG, et al (1987). A new member of the immunoglobulin superfamily—CTLA-4. Nature.

[B22] Brunner MC, Chambers CA, Chan FK, Hanke J, Winoto A, Allison JP (1999). CTLA-4-mediated inhibition of early events of T cell proliferation. J Immunol.

[B23] Tivol EA, Borriello F, Schweitzer AN, Lynch WP, Bluestone JA, Sharpe AH (1995). Loss of CTLA-4 leads to massive lymphoproliferation and fatal multiorgan tissue destruction, revealing a critical negative regulatory role of CTLA-4. Immunity.

[B24] Linsley PS, Greene JL, Brady W, Bajorath J, Ledbetter JA, Peach R (1994). Human B7-1 (CD80) and B7-2 (CD86) bind with similar avidities but distinct kinetics to CD28 and CTLA-4 receptors. Immunity.

[B25] van der Merwe PA, Bodian DL, Daenke S, Linsley P, Davis SJ (1997). CD80 (B7-1) binds both CD28 and CTLA-4 with a low affinity and very fast kinetics. J Exp Med.

[B26] Schwartz JC, Zhang X, Fedorov AA, Nathenson SG, Almo SC (2001). Structural basis for co-stimulation by the human CTLA-4/B7-2 complex. Nature.

[B27] Linsley PS, Bradshaw J, Greene J, Peach R, Bennett KL, Mittler RS (1996). Intracellular trafficking of CTLA-4 and focal localization towards sites of TCR engagement. Immunity.

[B28] Egen JG, Allison JP (2002). Cytotoxic T lymphocyte antigen-4 accumulation in the immunological synapse is regulated by TCR signal strength. Immunity.

[B29] Read S, Greenwald R, Izcue A, Robinson N, Mandelbrot D, Francisco L, et al (2006). Blockade of CTLA-4 on CD4+CD25+ regulatory T cells abrogates their function in vivo. J Immunol.

[B30] Freeman GJ, Long AJ, Iwai Y, Bourque K, Chernova T, Nishimura H, et al (2000). Engagement of the PD-1 immunoinhibitory receptor by a novel B7 family member leads to negative regulation of lymphocyte activation. J Exp Med.

[B31] Latchman Y, Wood CR, Chernova T, Chaudhary D, Borde M, Chernova I, et al (2001). PD-L2 is a second ligand for PD-1 and inhibits T cell activation. Nat Immunol.

[B32] Loke P, Allison JP (2003). PD-L1 and PD-L2 are differentially regulated by Th1 and Th2 cells. Proc Natl Acad Sci USA.

[B33] Kisielow M, Kisielow J, Capoferri-Sollami G, Karjalainen K (2005). Expression of lymphocyte activation gene 3 (LAG-3) on B cells is induced by T cells. Eur J Immunol.

[B34] Triebel F, Jitsukawa S, Baixeras E, Roman-Roman S, Genevee C, Viegas-Pequignot E, et al (1990). LAG-3, a novel lymphocyte activation gene closely related to CD4. J Exp Med.

[B35] Baixeras E, Huard B, Miossec C, Jitsukawa S, Martin M, Hercend T, et al (1992). Characterization of the lymphocyte activation gene 3-encoded protein. A new ligand for human leukocyte antigen class II antigens J Exp Med.

[B36] Huard B, Tournier M, Hercend T, Triebel F, Faure F (1994). Lymphocyte-activation gene 3/major histocompatibility complex class II interaction modulates the antigenic response of CD4+ T lymphocytes. Eur J Immunol.

[B37] Huard B, Mastrangeli R, Prigent P, Bruniquel D, Donini S, El-Tayar N, et al (1997). Characterization of the major histocompatibility complex class II binding site on LAG-3 protein. Proc Natl Acad Sci USA.

[B38] Souri Z, Wierenga AP, Mulder A, Jochemsen AG, Jager MJ (2019). HLA expression in uveal melanoma: An indicator of malignancy and a modifiable immunological target. Cancers (Basel).

[B39] Souri Z, Wierenga AP, van Weeghel C, van der Velden PA, Kroes WG, Luyten GP, et al (2019). Loss of BAP1 is associated with upregulation of the NFkB pathway and increased HLA class I expression in uveal melanoma. Cancers (Basel).

[B40] Souri Z, Jochemsen AG, Versluis M, Wierenga AP, Nemati F, van der Velden PA, et al (2020). HDAC inhibition increases HLA class I expression in uveal melanoma. Cancers (Basel).

[B41] Guy C, Mitrea DM, Chou PC, Temirov J, Vignali KM, Liu X, et al (2022). LAG3 associates with TCR-CD3 complexes and suppresses signaling by driving co-receptor-Lck dissociation. Nat Immunol.

[B42] Chung LY, Tang SJ, Wu YC, Sun GH, Liu HY, Sun KH (2015). Galectin-3 augments tumor initiating property and tumorigenicity of lung cancer through interaction with β-catenin. Oncotarget.

[B43] Wang J, Sanmamed MF, Datar I, Su TT, Ji L, Sun J, et al (2019). Fibrinogen-like protein 1 is a major immune inhibitory ligand of LAG-3. Cell.

[B44] Bae J, Lee SJ, Park CG, Lee YS, Chun T (2014). Trafficking of LAG-3 to the surface on activated T cells via its cytoplasmic domain and protein kinase C signaling. J Immunol.

[B45] Nagasaki J, Togashi Y (2022). A variety of ‘exhausted’ T cells in the tumor microenvironment. Int Immunol.

[B46] Banerjee S, Nahar U, Dahiya D, Mukherjee S, Dey P, Gupta R, et al (2022). Role of cytotoxic T cells and PD-1 immune checkpoint pathway in papillary thyroid carcinoma. Front Endocrinol (Lausanne).

[B47] Vaddepally RK, Kharel P, Pandey R, Garje R, Chandra AB (2020). Review of indications of FDA-approved immune checkpoint inhibitors per NCCN guidelines with the level of evidence. Cancers (Basel).

[B48] Korman AJ, Garrett-Thomson SC, Lonberg N (2022). The foundations of immune checkpoint blockade and the ipilimumab approval decennial. Nat Rev Drug Discov.

[B49] Guo Z, Zhang R, Yang AG, Zheng G (2023). Diversity of immune checkpoints in cancer immunotherapy. Front Immunol.

[B50] Kamali AN, Bautista JM, Eisenhut M, Hamedifar H (2023). Immune checkpoints and cancer immunotherapies: Insights into newly potential receptors and ligands. Ther Adv Vaccines Immunother.

[B51] Chalan P, Di Dalmazi G, Pani F, De Remigis A, Corsello A, Caturegli P (2018). Thyroid dysfunctions secondary to cancer immunotherapy. J Endocrinol Invest.

[B52] Kotwal A, Kottschade L, Ryder M (2020). PD-L1 inhibitor-induced thyroiditis is associated with better overall survival in cancer patients. Thyroid.

[B53] Zhou N, Velez MA, Bachrach B, Gukasyan J, Fares CM, Cummings AL, et al (2021). Immune checkpoint inhibitor induced thyroid dysfunction is a frequent event post-treatment in NSCLC. Lung Cancer.

[B54] Arnaud-Coffin P, Maillet D, Gan HK, Stelmes JJ, You B, Dalle S, et al (2019). A systematic review of adverse events in randomized trials assessing immune checkpoint inhibitors. Int J Cancer.

[B55] Hodi FS, O’Day SJ, McDermott DF, Weber RW, Sosman JA, Haanen JB, et al (2010). Improved survival with ipilimumab in patients with metastatic melanoma. N Engl J Med.

[B56] Motzer RJ, Rini BI, McDermott DF, Redman BG, Kuzel TM, Harrison MR, et al (2015). Nivolumab for metastatic renal cell carcinoma: Results of a randomized phase II trial. J Clin Oncol.

[B57] Robert C, Ribas A, Wolchok JD, Hodi FS, Hamid O, Kefford R, et al (2014). Anti-programmed-death-receptor-1 treatment with pembrolizumab in ipilimumab-refractory advanced melanoma: A randomised dose-comparison cohort of a phase 1 trial. Lancet.

[B58] Min L, Vaidya A, Becker C (2011). Thyroid autoimmunity and ophthalmopathy related to melanoma biological therapy. Eur J Endocrinol.

[B59] Topalian SL, Hodi FS, Brahmer JR, Gettinger SN, Smith DC, McDermott DF, et al (2012). Safety, activity, and immune correlates of anti-PD-1 antibody in cancer. N Engl J Med.

[B60] Topalian SL, Sznol M, McDermott DF, Kluger HM, Carvajal RD, Sharfman WH, et al (2014). Survival, durable tumor remission, and long-term safety in patients with advanced melanoma receiving nivolumab. J Clin Oncol.

[B61] Ryder M, Callahan M, Postow MA, Wolchok J, Fagin JA (2014). Endocrine-related adverse events following ipilimumab in patients with advanced melanoma: A comprehensive retrospective review from a single institution. Endocr Relat Cancer.

[B62] Robert C, Long GV, Brady B, Dutriaux C, Maio M, Mortier L, et al (2015). Nivolumab in previously untreated melanoma without BRAF mutation. N Engl J Med.

[B63] Larkin J, Chiarion-Sileni V, Gonzalez R, Grob JJ, Cowey CL, Lao CD, et al (2015). Combined nivolumab and ipilimumab or monotherapy in untreated melanoma. N Engl J Med.

[B64] Garon EB, Rizvi NA, Hui R, Leighl N, Balmanoukian AS, Eder JP, et al (2015). ; KEYNOTE-001 Investigators. Pembrolizumab for the treatment of non-small-cell lung cancer N Engl J Med.

[B65] Brahmer JR, Tykodi SS, Chow LQ, Hwu WJ, Topalian SL, Hwu P, et al (2012). Safety and activity of anti-PD-L1 antibody in patients with advanced cancer. N Engl J Med.

[B66] McDermott DF, Sosman JA, Sznol M, Massard C, Gordon MS, Hamid O, et al (2016). Atezolizumab, an anti–programmed death-ligand 1 antibody, in metastatic renal cell carcinoma: Long-term safety, clinical activity, and immune correlates from a phase I a study. J Clin Oncol.

[B67] Tawbi HA, Schadendorf D, Lipson EJ, Ascierto PA, Matamala L, Castillo Gutiérrez E, et al (2022). ; RELATIVITY-047 Investigators. Relatlimab and nivolumab versus nivolumab in untreated advanced melanoma N Engl J Med.

[B68] Sukik A, Mohamed S, Habib MB, Sardar S, Tanous B, Tahtouh R, et al (2020). The unusual late-onset graves’ disease following Hashimoto’s related hypothyroidism: A case report and literature review. Case Rep Endocrinol.

[B69] Bartalena L, Piantanida E, Gallo D, Ippolito S, Tanda ML (2022). Management of Graves’ hyperthyroidism: Present and future. Expert Rev Endocrinol Metab.

[B70] Nabi M, Noor R, Zahid A, Zulfiqar T, Khalid A, Ri S (2022). Grave’s disease: Pathophysiology of a model autoimmune disease. Arch Microbiol.

[B71] Ueda H, Howson JM, Esposito L, Heward J, Snook H, Chamberlain G, et al (2003). Association of the T-cell regulatory gene CTLA4 with susceptibility to autoimmune disease. Nature.

[B72] Han S, Zhang S, Zhang W, Li R, Li Y, Wang Z, et al (2006). CTLA4 polymorphisms and ophthalmopathy in Graves’ disease patients: association study and meta-analysis. Hum Immunol.

[B73] Takahashi M, Kimura A (2010). HLA and CTLA4 polymorphisms may confer a synergistic risk in the susceptibility to Graves’ disease. J Hum Genet.

[B74] Borodic G, Hinkle DM, Cia Y (2011). Drug-induced graves disease from CTLA-4 receptor suppression. Ophthalmic Plast Reconstr Surg.

[B75] Azmat U, Liebner D, Joehlin-Price A, Agrawal A, Nabhan F (2016). Treatment of ipilimumab induced Graves’ disease in a patient with metastatic melanoma. Case Rep Endocrinol.

[B76] Gan EH, Mitchell AL, Plummer R, Pearce S, Perros P (2017). Tremelimumab-induced Graves hyperthyroidism. Eur Thyroid J.

[B77] Brancatella A, Viola N, Brogioni S, Montanelli L, Sardella C, Vitti P, et al (2019). Graves’ disease induced by immune checkpoint inhibitors: A case report and review of the literature. Eur Thyroid J.

[B78] Yamada H, Okajima F, Onda T, Fujimori S, Emoto N, Sugihara H (2020). New-onset graves’ disease after the initiation of nivolumab therapy for gastric cancer: A case report. BMC Endocr Disord.

[B79] Moore GH, Rootman DB (2016). Orbital inflammatory disease management. Expert Rev Ophthalmol.

[B80] Ludgate M, Baker G (2002). Unlocking the immunological mechanisms of orbital inflammation in thyroid eye disease. Clin Exp Immunol.

[B81] Bawazeer A, Rahali W, Alsharif A, Alshehri M, Maksood L, Babkier A, et al (2023). Idiopathic orbital inflammation treated with rituximab monotherapy. Cureus.

[B82] Srivastava A, Al-Zubidi N, Appelbaum E, Gombos DS, Nader ME, Gidley PW, et al (2020). Immune-related oral, otologic, and ocular adverse events. Adv Exp Med Biol.

[B83] Gan L, Chen H, Liu X, Zhang L (2023). Ophthalmic immune-related adverse events associated with immune checkpoint inhibitors. Front Immunol.

[B84] Mohammadi P, Hesari M, Chalabi M, Salari F, Khademi F (2022). An overview of immune checkpoint therapy in autoimmune diseases. Int Immunopharmacol.

[B85] Bahn RS (2010). Graves’ ophthalmopathy. N Engl J Med.

[B86] Bartalena L, Fatourechi V (2014). Extrathyroidal manifestations of Graves’ disease: A 2014 update. J Endocrinol Invest.

[B87] Chin YH, Ng CH, Lee MH, Koh JW, Kiew J, Yang SP, et al (2020). Prevalence of thyroid eye disease in Graves’ disease: A meta-analysis and systematic review. Clin Endocrinol (Oxf).

[B88] Ueland HO, Neset MT, Methlie P, Ueland GÅ, Pakdel F, Rødahl E

[B89] Tanda ML, Piantanida E, Liparulo L, Veronesi G, Lai A, Sassi L, et al (2013). Prevalence and natural history of Graves’ orbitopathy in a large series of patients with newly diagnosed graves’ hyperthyroidism seen at a single center. J Clin Endocrinol Metab.

[B90] Hiromatsu Y, Eguchi H, Tani J, Kasaoka M, Teshima Y (2014). Graves’ ophthalmopathy: Epidemiology and natural history. Intern Med.

[B91] Ionescu IC, van Trotsenburg PA, Paridaens D, Tanck M, Mooij CF, Cagienard E, et al (2022). Pediatric Graves’ orbitopathy: A multicentre study. Acta Ophthalmol.

[B92] Kahaly GJ, Petrak F, Hardt J, Pitz S, Egle UT (2005). Psychosocial morbidity of Graves’ orbitopathy. Clin Endocrinol (Oxf).

[B93] Potvin AR, Pakdel F, Saeed P

[B94] Kashkouli MB, Alemzadeh SA, Aghaei H, Pakdel F, Abdolalizadeh P, Ghazizadeh M, et al (2018). Subjective versus objective dry eye disease in patients with moderate-severe thyroid eye disease. Ocul Surf.

[B95] Pakdel F, Sullivan TJ, Pirmarzdashti N (2022). Pathophysiology of autoimmune orbital diseases and target therapy for orbital inflammatory and neoplastic diseases. Transl Autoimmun.

[B96] Bartalena L, Baldeschi L, Dickinson AJ, Eckstein A, Kendall-Taylor P, Marcocci C, et al (2008). Consensus statement of the European group on Graves’ orbitopathy (EUGOGO) on management of Graves’ orbitopathy. Thyroid.

[B97] Kashkouli MB, Heidari I, Pakdel F, Jam S, Honarbakhsh Y, Mirarmandehi B (2011). Change in quality of life after medical and surgical treatment of graves’ ophthalmopathy. Middle East Afr J Ophthalmol.

[B98] Bahmani-Kashkouli M, Pakdel F, Astaraki A, Hashemi M, Honarbakhsh Y, Mirarmandehi B, et al (2009). Quality of life in patients with thyroid eye disease. J Ophthalmic Vis Res.

[B99] Kashkouli MB, Kaghazkanani R, Heidari I, Ketabi N, Jam S, Azarnia S, et al (2011). Bilateral versus unilateral thyroid eye disease. Indian J Ophthalmol.

[B100] Pakdel F, Ghazavi R, Heidary R, Nezamabadi A, Parvizi M, Haji Safar Ali Memar M, et al (2019). Effect of selenium on thyroid disorders: Scientometric Analysis. Iran J Public Health.

[B101] Barrio-Barrio J, Sabater AL, Bonet-Farriol E, Velázquez-Villoria Á, Galofré JC (2015). Graves’ ophthalmopathy: VISA versus EUGOGO classification, assessment, and management. J Ophthamol.

[B102] Ban Y, Davies TF, Greenberg DA, Kissin A, Marder B, Murphy B, et al (2003). Analysis of the CTLA-4, CD28, and inducible costimulator (ICOS) genes in autoimmune thyroid disease. Genes Immun.

[B103] Goyal I, Pandey MR, Sharma R, Chaudhuri A, Dandona P (2021). The side effects of immune checkpoint inhibitor therapy on the endocrine system. Indian J Med Res.

[B104] McElnea E, Ní Mhéalóid Á, Moran S, Kelly R, Fulcher T (2014). Thyroid-like ophthalmopathy in a euthyroid patient receiving Ipilimumab. Orbit.

[B105] Dalvin LA, Shields CL, Orloff M, Sato T, Shields JA (2018). Checkpoint inhibitor immune therapy: Systemic indications and ophthalmic side effects. Retina.

[B106] Liu Z, Liu Y, Liu M, Gong Q, Shi A, Li X, et al (2022). PD-L1 inhibits T cell-induced cytokines and hyaluronan expression via the CD40-CD40L pathway in orbital fibroblasts from patients with thyroid associated ophthalmopathy. Front Immunol.

[B107] Berrih-Aknin S, Frenkian-Cuvelier M, Eymard B

[B108] Dresser L, Wlodarski R, Rezania K, Soliven B (2021). Myasthenia gravis: Epidemiology, pathophysiology and clinical manifestations. J Clin Med.

[B109] Virameteekul S, Charoensri S, Sawanyawisuth K, Tiamkao S (2019). Concurrence of myasthenia gravis and thyroid disorders: A retrospective database study. J ASEAN Fed Endocr Soc.

[B110] Amin S, Aung M, Gandhi FR, Pena Escobar JA, Gulraiz A, Malik BH (2020). Myasthenia gravis and its association with thyroid diseases. Cureus.

[B111] Masood I, Yasir M, Aiman A, Kudyar RP (2009). Autoimmune thyroid disease with myasthenia gravis in a 28-year-old male: A case report. Cases J.

[B112] Mangaraj S, Choudhury AK, Mohanty BK, Baliarsinha AK (2016). Neurological manifestations of Graves’ disease: A case report and review of the literature. J Neurosci Rural Pract.

[B113] Salhi H, Ajdi F (2019). [Hypothyroidism and myasthenia: A case study]. Pan Afr Med J.

[B114] Becquart O, Lacotte J, Malissart P, Nadal J, Lesage C, Guillot B, et al (2019). Myasthenia gravis induced by immune checkpoint inhibitors. J Immunother.

[B115] Suzuki S, Ishikawa N, Konoeda F, Seki N, Fukushima S, Takahashi K, et al (2017). Nivolumab-related myasthenia gravis with myositis and myocarditis in Japan. Neurology.

[B116] Maeda O, Yokota K, Atsuta N, Katsuno M, Akiyama M, Ando Y (2016). Nivolumab for the treatment of malignant melanoma in a patient with pre-existing myasthenia gravis. Nagoya J Med Sci.

[B117] Dugena O, Zheng C, Taylor J, Wong A (2022). Pembrolizumab-induced myasthenia gravis: literature review of ocular manifestations and a refractory case. J Immunother.

[B118] Kamo H, Hatano T, Kanai K, Aoki N, Kamiyama D, Yokoyama K, et al (2019). Pembrolizumab-related systemic myositis involving ocular and hindneck muscles resembling myasthenic gravis: A case report. BMC Neurol.

[B119] Liu Q, Ayyappan S, Broad A, Narita A (2019). Pembrolizumab-associated ocular myasthenia gravis. Clin Exp Ophthalmol.

[B120] Michels KL, Karagianis Do AG, Simon SS (2019). Pembrolizumab-associated diplopia secondary to idiopathic orbital inflammatory syndrome. J Clin Ophthalmol.

[B121] Lorenzo CJ, Fitzpatrick H, Campdesuner V, George J, Lattanzio N (2020). Pembrolizumab-induced ocular myasthenic crisis. Cureus.

[B122] Garcez D, Clara AI, Moraes-Fontes MF, Marques JB (2022). A Challenging case of eyelid ptosis and diplopia induced by pembrolizumab. Cureus.

[B123] Jeyakumar N, Etchegaray M, Henry J, Lelenwa L, Zhao B, Segura A, et al (2020). The terrible triad of checkpoint inhibition: A case report of myasthenia gravis, myocarditis, and myositis induced by cemiplimab in a patient with metastatic cutaneous squamous cell carcinoma. Case Reports Immunol.

[B124] Garibaldi M, Calabrò F, Merlonghi G, Pugliese S, Ceccanti M, Cristiano L, et al (2020). Immune checkpoint inhibitors (ICIs)-related ocular myositis. Neuromuscul Disord.

[B125] Tian CY, Ou YH, Chang SL, Lin CM (2021). Pembrolizumab-induced myasthenia gravis-like disorder, ocular myositis, and hepatitis: A case report. J Med Case Rep.

[B126] Jebaraj AP, Etheridge TJ, Winegar BA, Marx DP

